# Neural changes in the primate brain correlated with the evolution of complex motor skills

**DOI:** 10.1038/srep31084

**Published:** 2016-08-08

**Authors:** Y. Yamazaki, K. Hikishima, M. Saiki, M. Inada, E. Sasaki, R. N. Lemon, C. J. Price, H. Okano, A. Iriki

**Affiliations:** 1 Advanced Research Centers, Keio University, 35 Shinanomachi, Shinjuku-ku, Tokyo 160-8582, Japan; 2 Laboratory for Symbolic Cognitive Development, RIKEN Brain Science Institute, 2-1 Hirosawa, Wako, Saitama 351-0198, Japan; 3 Department of Physiology, Keio University School of Medicine, 35 Shinanomachi, Shinjuku-ku, Tokyo 160-8582, Japan; 4 Central Institute for Experimental Animals, 3-25-12 Tonomachi, Kawasaki-ku, Kawasaki, Kanagawa 210-0821, Japan; 5 Sobell Department of Motor Neuroscience and Movement Disorders, UCL Institute of Neurology, London WC1N 3BG, UK; 6 Wellcome Trust Centre for Neuroimaging, University College London, London WC1N3BG, UK; 7 Laboratory for Marmoset Neural Architecture, RIKEN Brain Science Institute, 2-1 Hirosawa, Wako, Saitama 351-0198, Japan

## Abstract

Complex motor skills of eventual benefit can be learned after considerable trial and error. What do structural brain changes that accompany such effortful long-term learning tell us about the mechanisms for developing innovative behavior? Using MRI, we monitored brain structure before, during and after four marmosets learnt to use a rake, over a long period of 10–13 months. Throughout learning, improvements in dexterity and visuo-motor co-ordination correlated with increased volume in the lateral extrastriate cortex. During late learning, when the most complex behavior was maintained by sustained motivation to acquire the skill, the volume of the nucleus accumbens increased. These findings reflect the motivational state required to learn, and show accelerated function in higher visual cortex that is consistent with neurocognitive divergence across a spectrum of primate species.

The behavioral repertoire of a particular species evolves to match the environmental demands through adaptation of bodily morphologies and their functions, controlled by the central nervous system. Novel tool use, for example, can be acquired even after considerable trial and error^1^,^2^,^3^. This requires sustained motivation to learn^4^, and changes in brain structure as well as function. Here we investigate the changes in brain anatomy that correlated with sustained motivation over the course of a whole year when common marmosets were learning to use a tool to carry out a demanding task^5^. The training involved several thousands of trials that became progressively more complex (Fig. 1a). There were no physical constraints or food control before training and the amount of food reward per trial was kept constant. This suggests that their learning was not weakened by repeated failures, but was reinforced by a finite food reward eventually earned through effortful challenges.

Changes in adult marmoset brains during the long-term training were measured with structural MRI. Early in training, we expected changes in the control of visually guided movement. We expected that changes in the later training phases, when the complexity of learning increased, would reflect the higher levels of motivation required relative to the early phases. The effect of learning in later versus earlier phases was tested by using four different learning stages that systematically increased the degree to which the tool had to be manipulated to retrieve the food. Thus, we searched for brain changes that (i) occurred early and increased in proportion to motor control abilities; (ii) occurred late when the skillful but effortful movements were reinforced.

## Results

### Acquisition of tool use behavior

**Figure 1:**
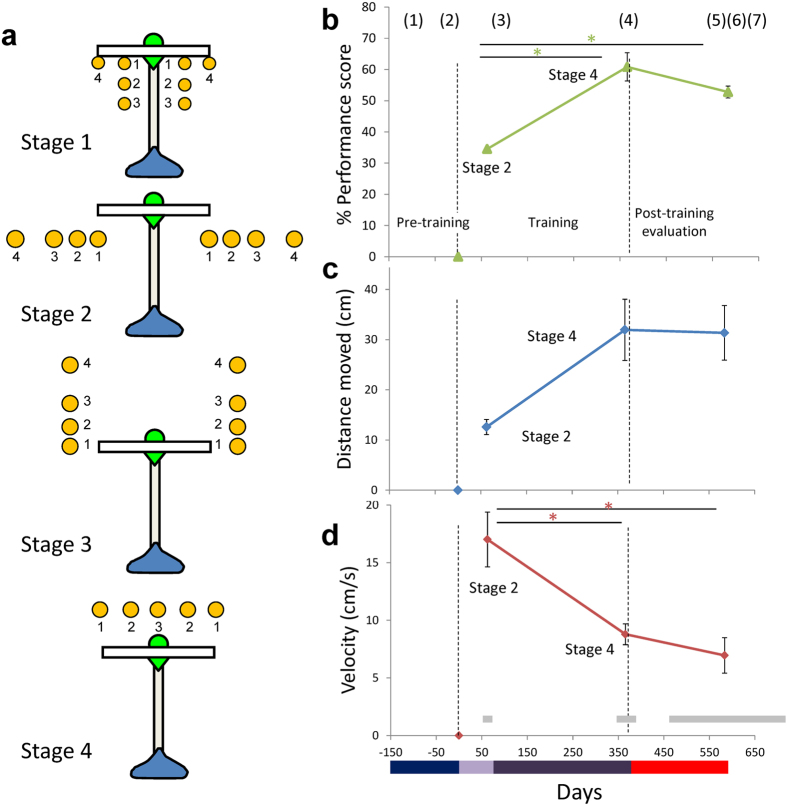
Task protocol and behavioral indices for tool use by common marmosets. **(a)** Top view of the tool showing the head (white), the shaft (gray) and the handle (blue) in the conditions in each training stage. Position of food items is indicated by small yellow circles. The small numbers (1–4) in each Stage denote the steps within a Stage. **(b–d)** Behavioral indices for tool use by common marmosets, averaged for four marmosets with standard errors of means (SE), as a function of experimental phases depicted in days (see Fig. S1 for individual data). Numbers (1 to 7) in parenthesis on the top of (**b**) show the time points at which MRI scans were carried out. Horizontal gray bars in (**d**) indicated the ranges in days at which the scanning was carried out in individual subjects. A color bar on the bottom of the figures corresponds to the experimental phases of pre-learning evaluation (dark blue), Stage 1 to 2 (light purple), Stage 3 to 4 (dark purple), and post-learning evaluation (red). Significant differences found by multiple comparison analysis were depicted in *(p < 0.05). **(b)** % performance score. 100% means the marmosets could retrieve the food item without any help from the experimenter in all tested trials (Methods). **(c)** The distance moved (cm) by the rake head in each trial. **(d)** The mean velocity (cm/s) of the rake head during manipulation of the tool.

We trained four adult female common marmosets to use a rake-shaped tool to retrieve food through the protocol established previously^5^ (see Methods). Briefly, the learning process was divided into four stages (Stages 1 to 4), each of which consisted of three to four minor steps (Fig. 1a). Hereafter we describe Stage 1 step 1, for example, as “Stage 1–1”. Because the marmosets were free from any food and water control, and participation in the task was totally dependent on the subjects’ own motivation, the number of executed trials per session varied considerably from subject to subject throughout the protocol. All subjects, however, successfully achieved the final step of Stage 4–3, which replicated our previous results^5^. The number of trials (actual number of days in which marmosets participated and the total duration of the learning period, in months) required to finish all the training stages were 4941 (158 days in 10 months), 3159 (150 days in 13 months), 5067 (150 days in 10 months), 3648 (174 days in 13 months) for subjects 36, 54, 66, and 70, respectively. The faster learners (54 and 70) therefore engaged in 1000 trials less than the slower learners (36 and 66).

Even after long periods of learning, the marmosets achieved at most around 60% proficiency on the task (Fig. 1b). However, performance scores continued to increase in the later stage of learning (from Stage 2–4 to 4–3) and were maintained until the post-learning period (Post). This was confirmed by a one way ANOVA, with 3 time points: 2–4, 4–3 and post (F (2, 9) = 22.548, p = 0.0003). Following correction for multiple comparisons using Tukey’s HSD, post hoc tests showed significant changes between Stages 2–4 and 4–3 (p = 0.0004); and Stages 2–4 and Post (p = 0.0004), with no significant difference between Stages 4–3 and post (p = 0.166).

The distance moved by the rake head in each trial was greater in Stage 4 than in any of the preceding stages (Fig. 1c). This was true for all subjects except 70. Across subjects, the difference across task stages was just short of significant (F (2, 9) = 4.067, p = 0.055). However, high correlations between distance moved and learning days were found in 54 (0.992) and 70 (0.903) but not in 36 (0.154) and 66 (0.445).

The mean velocity of the rake head during manipulation of the tool declined gradually over the course of the learning period (Fig. 1d), which was the opposite to the trend shown by Japanese macaques^6^, but was consistent with our previous marmoset study^5^. There was a significant effect of stage (one-way ANOVA, F (2, 9) = 9.735, p = 0.006) together with a significant difference between Stage 2–4 and 4–3 (Tukey’s HSD test, p = 0.020), and between Stage 2–4 and Post (p = 0.006). As with the performance score, there was no significant difference between Stage 4–3 and Post (p = 0.738).

### Gray matter changes correlated with behavioral indexes

We identified three prominent areas of gray matter volume increase that correlated with higher performance scores or the rake distance moved (p < 0.05 after correction for multiple comparisons across the whole brain at the voxel level (familywise error (FWE) corrected): (i) the lateral extrastriate cortex (V2/V3), which are likely to reflect increased visuomotor control abilities in earlier stages; (ii) nucleus accumbens (Acb), which are likely to reflect strengthened reinforcing effects of effortful tool use in late learning stages; and (iii) the superior colliculus (SC), which are likely to reflect increased visual spatial attention to the food item and the tool. All volumetric increases in brain structure were observed bilaterally, and were confirmed in individual analyses of at least 2 subjects (Table 1).

### Left and right nucleus accumbens

**Figure 2:**
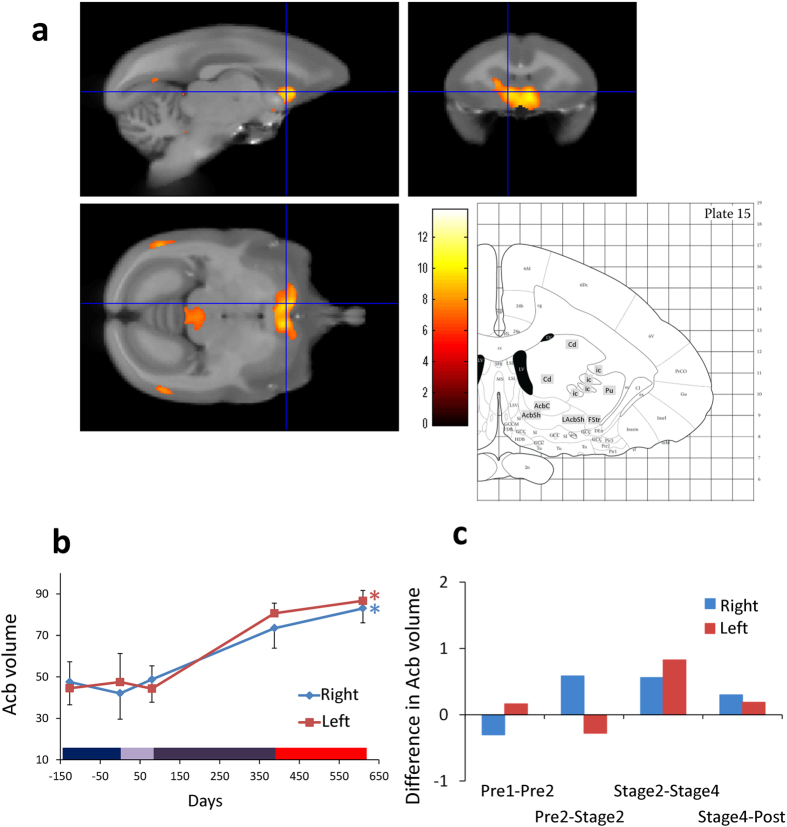
Gray matter increases in bilateral Acb. **(a)** The color scale indicates the t score and the area of significance has been superimposed on a template brain, together with the corresponding coronal image (interaural −11.8−mm, Plate 15) of the marmoset brain atlas^7^. AcbC: accumbens nucleus core; AcbSh: accumbens nucleus shell; LAcbSh: accumbens nucleus shell, lateral part; Cd: caudate nucleus; FStr: fundus of striatum; ic: internal capsule; Pu: putamen. **(b)** Time course of bilateral gray matter volume change in peak voxels in Acb, depicted in right (blue) and left (red) hemispheres, averaged across four subjects, with SE (n = 4, *p < 0.05). Color bar on the bottom is the same as in Fig. 1. **(c)** Difference in bilateral Acb gray matter volume in peak voxels in both hemispheres between adjacent periods, calculated per week. Left gray matter volume increased especially later phases of the experiment.

The location, time course and statistics related to Acb volume changes (i.e. change in the number of voxels identified as Acb region) in the later phase of learning are detailed in Fig. 2a,b, together with the coronal images corresponding to the same coordinates in the stereotaxic brain atlas^7^. The rate of volume change per week was higher for later than earlier learning (Fig. 2c).

A one-way ANOVA with 3 time points indicated significant effects in both the right and left hemisphere (F (4, 15) = 3.063, p = 0.0496 in right; and 4.237, p = 0.017 in left). In the later phases of learning (between Stages 2 and 4), Acb volume changes were located in xyz standard space at [0.7, 9.0, 7.2] in the right hemisphere and [−1.3, 10.6, 9.3] in the left hemisphere. These effects were highly significant, Z scores = 6.24 in the right, 5.83 in the left; p < 0.05 after FWE correction for multiple comparisons.

Individual data (Table 1 and Fig. S2a) confirmed that, in both left and right Acb, volume increased for all subjects in the later learning stages. Interestingly, the effect sizes were largest in the faster learners (54 and 70). In each of these animals, the correlation between Acb (bilateral) volume and distance moved of the rake head reached significance (p < 0.05). The correlation between left Acb volume and performance score also reached significance in subject 54 (p < 0.05, FWE corrected).

### Changes in the visual cortex

**Figure 3:**
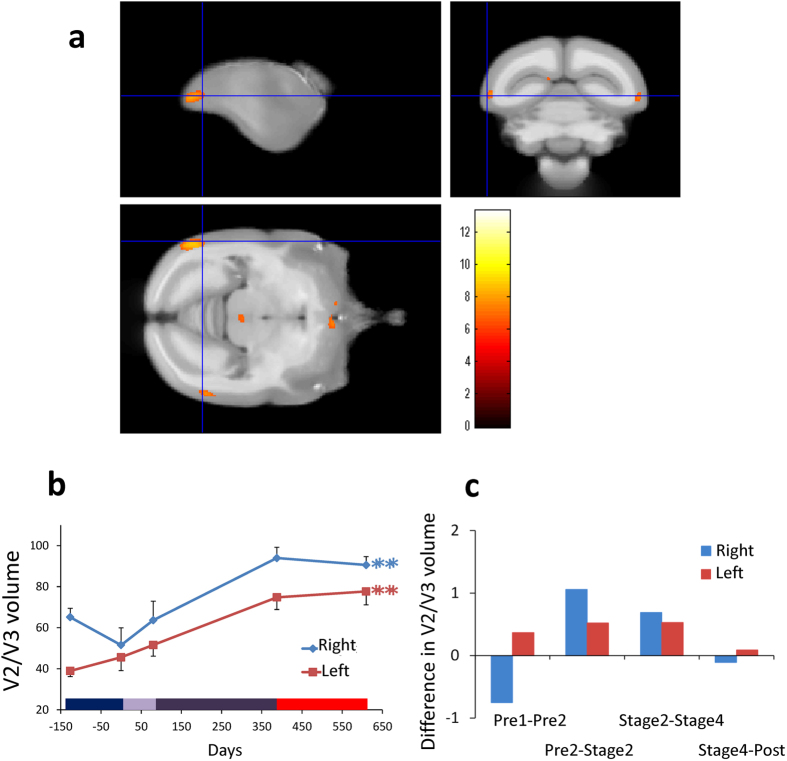
Gray matter increases in bilateral lateral extrastriate cortex (V2/V3). **(a)** Bilateral gray matter increases in visual cortical area V3 and in left V2 as a function of experimental phases. **(b)** Time course of averaged gray matter change in peak voxels in ROI in bilateral V3, with SE (n = 4, **p < 0.01). Gray matter volume increased dramatically across the different training phases. Color bar on the bottom is the same as in Fig. 1. **(c)** Difference in bilateral V3 gray matter volume in both hemispheres between the adjacent periods, calculated per week. See also Fig. S2.

The early and late gray matter changes in visual cortex were observed in the second visual area (lateral extrastriate cortex (V2/V3), Fig. 3a,b), together with middle temporal area (MT^8^,^9^, observed only in subject 54, see Fig. S3). The rate of volume change per week (Fig. 3c) confirms that they occurred during early and late learning but not before or after learning. All four subjects showed volume increases in V3 as tool performance improved (Table 1 and Fig. S2b) with this effect reaching significance in one of the slower learners (66) in left V2/V3.

The location of these effects in the x,y,z coordinate system of the stereotaxic brain atlas^7^, were [9.8, −5.0, 9.0] in right V3, [−9.4, −7.6, 8.6] in left V3, and [−8.4, −8.4, −7.0] in left V2. The corresponding Z scores were 5.1, 5.79 and 6.21; n = 4; p < 0.05; FWE corrected. The statistics related to the overall effect of time course (see Fig. 3b) were: F (4, 15) = 7.687, p = 0.001 in the right hemisphere and F (4, 15) = 9.805, p = 0.0004 in the left hemisphere.

**Figure 4:**
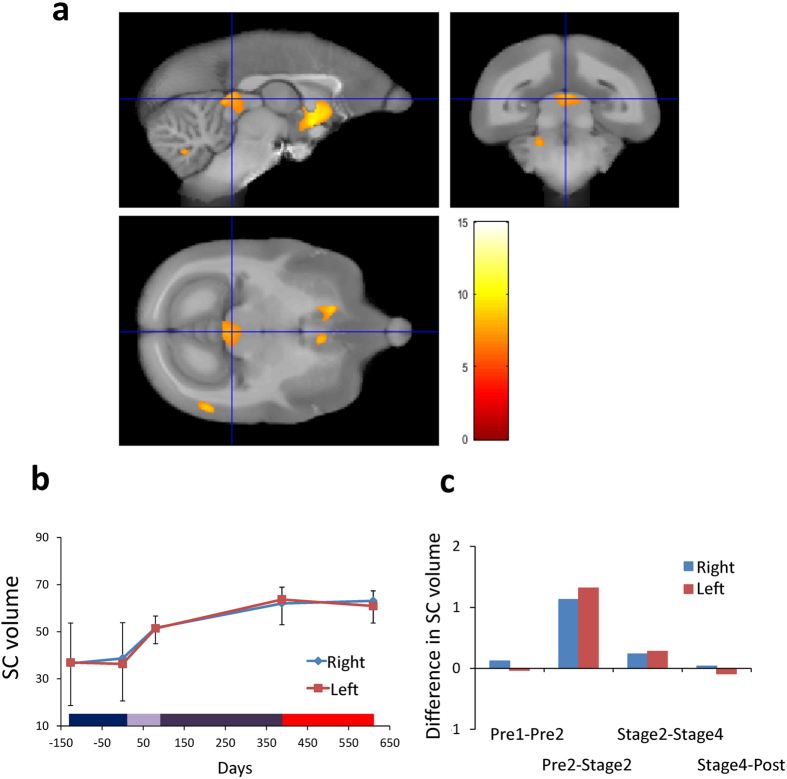
Gray matter increases in the lateral superior colliculi (SC). **(a)** Bilateral gray matter increases in SC as a function of experimental phases. **(b)** Time course of averaged gray matter change in peak voxels in ROI in bilateral SC, with SE (n = 4, n.s.). Color bar on the bottom is the same as in Fig. 1. **(c)** Difference in bilateral SC gray matter volume in both hemispheres between the adjacent periods, calculated per week.

The cluster of red voxels in the brain stem in Fig. 4a corresponds to bilateral SC, especially its intermediate gray matter:[0.3, −0.7, 10.9] on the right, and [−0.5, −0.5, 10.9] on the left. The corresponding Z scores were 5.68 and 5.64, respectively (n = 4; p < 0.05; FWE corrected). The statistics related to the overall effect of time course (see Fig. 4b) were: F (4, 15) = 0.910, p = 0.483 in the right hemisphere and F (4, 15) = 1.220, p = 0.344 in the left hemisphere.

## Discussion

Intensive behavioral training has been reported to change brain volume in areas involved in the trained task^6^. The underlying mechanisms have been associated with increased neuronal activity^10^ and enhanced connectivity between different parts of the cortex^11^. In this study, we demonstrated for the first time that Acb volume increased bilaterally in the later learning stages (Fig. 2). As task complexity increased as the training proceeded, motivation to use the tool must also increase, because the amount of reward obtained per trial did not change. The association of Acb with reward is consistent with previous studies that reported activation in Acb when the effort to get a reward increased^12^, and when subjects learnt to perform a task skillfully^13^. In macaques, the functional connectivity between primary motor area (M1) and Acb^14^, and activity of Acb^15^, was strengthened as they became more skillful in picking up food items. Thus, the increased volume of Acb in the current study could be related to the positive engagement in an effortful task that was necessary to obtain reward. In humans, this is referred to as intrinsic motivation and is said to be critical for learning demanding tasks^4^,^16^. In fact, the greater increase in Acb gray matter signal observed in faster compared to slower learners would suggest the faster learners had greater motivation to use the tool.

Because a change in the Acb was not found to be associated with rapid learning of tool use by macaques^6^ or with juggling in human subjects^17^, it could be related to specific characteristics of the marmoset’s long term learning procedure, which involved gradual, self-paced, step-by-step challenges (SI2).

In the occipital lobe we found bilateral increases in gray matter volume in lateral extrastriate cortex (V2/V3, Fig. 3). In one subject (54), a region anterior to V2/V3 was also affected, which may have included area MT^8^,^9^ (Fig. S3). Change in the lateral extrastriate cortex may be related to the fact that in the early learning stage the subjects were fixating attentively on the food items and/or their hands that were holding the tool. This is consistent with the role of visual cortical areas in cue-invariant perception of moving objects^18^,^19^. In this study, the marmosets freely observed the tool and the food item from a variety of body positions, distances and angles as they moved around the cage, which is different from our Japanese macaque study in which no volumetric increase in visual areas were reported^6^. Of course, there are major differences in corticospinal control of the hand in marmosets versus macaques^20^.

Interestingly, the V2-V3-MT complex is expanded in primates^21^, and is assumed to represent evolutionary newer visual areas, gradually developing from the evolutionary older areas, MT and primary visual area (V1), into higher-order visual areas^22^. During ontogeny, these areas mature when finer visuotopic mapping is acquired in later developmental stages^21^. They may also be particularly sensitive to activity-dependent changes even in the adult brain. The volume increases we observe in these areas may therefore be related to accelerated function of the higher visual areas in lower primates following long-term exposure to a novel behavioral experience.

Significant volume changes were also observed in bilateral SC, especially its intermediate gray region (Fig. 4a). The activity of neurons in this area is related to orienting movements and selecting stimuli. Connections from this area to parietal cortex through thalamic nuclei, are thought to contribute to spatial attention functions associated with lateral intraparietal cortex^23^. The change in SC was most prominent in the early phase of the training (Fig. 4c) but the interaction between brain volume change and time did not reach significance. Thus, volume change in SC is better explained by the general characteristics of the tool use task, i.e. repeated spatial attention to the food items and movement of the tip of the tool when the animals were trying to retrieve food.

Another important observation found in the current study was that brain volume did not change in the months after tool training was complete. The final MRI scans were acquired 159 to 327 days after the training ended (see red horizontal bar on the x axis in Figs 2, 3, 4). This provides further evidence that the structural changes were related to the training. Moreover, brain structure remained stable rather than reversing. This stands in sharp contrast to the results obtained from macaques where we found some reversal of the volume 15 days after the end of the tool training^6^.

We are reporting the results of a within-subject longitudinal study with MRI measurements before and after training. The close correlation of brain changes with the onset and offset of training, followed by stability in brain structure months after training, provide strong evidence that volume changes were training related. However, the images analyzed with VBM involve comparing anatomical images of MRI, so we cannot say anything about the boundaries and the cytoarchitecture of the regions where volume changes were detected. Thus, the accuracy and interpretation of VBM data needs to be verified by histochemical analysis of the brain tissue^24^.

In this study, we employed a within-subject design to search for any structural changes in the brain associated with training, avoiding the possibility that group average changes might be obscured by individual differences. On the other hand, without an age-matched control group, the question may arise that the observed changes in brain structure reflected the normal developmental changes in marmosets around the age we chose to study. However, a recent longitudinal MRI study after birth in marmosets detected maximum brain volume at around nine months of age, followed by a gradual decrease until 18 months and being stable thereafter^25^. Because scans of the youngest subject (70) in this study started at 15 months and training at 19 months, this argues strongly in support of our argument that the increases detected were due to behavioral training, but not to normal development.

In conclusion, we have provided new insights into brain plasticity in adult primates by showing that a gradual and self-motivated acquisition of a novel behavior was associated with structural changes which may be related to the motivational state of the subjects when engaging in the task. We suggest that changes in Acb reflect an important role in maintaining the marmosets’ motivation to learn the novel behavior. The sustained engagement in the task also involved changes in brain structure uniquely developed in primates (higher visual areas, V2-V3-MT). Overall, our data suggest possible mechanisms for the development of innovative behavior, maintained by positive reinforcement of the newly acquired behavior, and stabilized by the structural brain changes as a result of learning. Such mechanisms lie at the heart of the neurocognitive divergence of the primate species^26^, and, in particular, the persistent and patient characteristics of the marmoset’s behavior in the wild^27^,^28^.

## Methods

### Subjects

Four experimentally naïve female marmosets were used in this study (age: 1y3m-2y7m old at the starting point). All animals were housed individually (63.5 (h) × 43.0 (w × 63.5 (d) cm) in a breeding room (12-h light–dark cycle, on average 28 °C and 50% of humidity). Testing sessions were always conducted during the daytime (10:00–15:00). The animals were fed regularly, and water was freely available in their cages.

### Ethics Statements

This study was approved by the Animal Experiment Committees in RIKEN Brain Science Institute and Central Institute for Experimental Animals, and was conducted in accordance with the Guidelines for Conducting Animal Experiments of RIKEN Brain Science Institute and of Central Institute for Experimental Animals.

### Apparatus

The subjects were carried into a test chamber (42.0 (h) × 34.0 (w) × 35.0 (d) cm), attached to a table (9.5 (h) × 35.0 (w) × 24.0 (d) cm). They were required to extend their arm through an aperture (2.0 (h) × 26.0 (w) cm, with 2.0 × 5.0 cm teeth) and to use a rake to retrieve a food item (0.5 cm sweet jelly). The rake had an acrylic head (2.5 cm (h) × 4.0 (w)) mounted on an aluminum shaft (7.0 cm long). The shaft adjacent to the rake head was painted in light green so that it could be detected easily by tracking software (Ethovision, Noldus), allowing measurement of the distance travelled and velocity of the rake head. During the experimental sessions, the subject’s behavior was recorded using video cameras (HDR-HC9, Sony). To encourage learning by the experimental subject, a companion marmoset was placed near the experimental animal in a second cage (27.5 (h) × 23.0 (w) × 42.0 (d) cm) located so that it could see the experimental marmoset.

### Experimental periods

#### Pre-learning evaluation period

There were three main phases in the present study: pre-learning evaluation period, learning, and post-learning evaluation period. In the pre-learning period, the interaction between marmosets and experimenters involved just handling and transferring them in a carrying cage when body weight was taken once per week. No other behavioral programs were conducted during this phase. The pre-learning evaluation period lasted for four months.

#### Learning period

We employed a protocol used in the previous study (Fig. 1a)^5^, except that the criterion for completion of each step in the protocol was set at three consecutive successful trials, instead of five trials, without any help from the experimenter. A trial started when the experimenter placed the tool and the food item on the table according to a given condition, and it lasted until the subject retrieved the food item and ate it. A successful trial was defined as the retrieval of the food item by the animals without any help from the experimenter. Learning was controlled over four stages, each of which consisted of three to four minor steps (indicated by the number near the circles representing the food in Fig. 1a). For each step there was a unique spatial relationship between the rake and the food item. In Stage 1, the food item was positioned just inside the rake head to induce any incidental pull on the rake handle. In Stage 2, the food was positioned to the left or the right of the rake to induce deliberate movements of the rake to the left or right. In Stage 3, the food was also positioned to the left or right of the rake but at increasing distances from the subject to induce new pushing movements. In Stage 4, the last stage, the food was positioned behind the rake so that the rake had to be rotated before being pushed behind the food. Changes in brain structure were measured at two key stages: the completion of Stage 2 relative to the pre-learning baseline (i.e. changes related to early learning) and the completion of Stage 4 relative to Stage 2 (i.e. changes related to late learning).

Sessions were conducted daily, 5 days per week. During the entire learning period, subjects could freely choose which hand/arm to use (see Tables S1 and SI 3). Additionally, they could participate in the session or decline to do so whenever they wanted to. Subjects typically used either hand except for the last step (Stage 4–3), in which the rake head and the food item were placed symmetrically in a straight line (see Fig. 1a).

#### Post-learning period

At the end of the study, subjects were no longer given the tool, and for five to ten months they returned to the same conditions as in the pre-learning period during which they never touched the tool nor went to the experimental room. After this post-learning period, tool use performance was evaluated for 5 daily sessions, during each of which 10 trials were conducted. To minimize any further training effect on the evaluation trials, the experimenter terminated a trial by giving the food item and moving on to the next trial if the marmoset failed to retrieve the food item on the second attempt in a trial. A trial in which the animals did not respond to the tool for more than a minute was excluded from the analysis. The final MRI scans were performed 7 to 31days after the final performance evaluation.

### Behavioral Scoring

We evaluated the tool use performance acquired by calculating the following indices: performance score, distance of the tool head moved, and averaged velocity of the rake head during tool use (Figs 1b–d and S1).

To evaluate the stabilization of tool use skill, we calculated the performance score in sessions just before the MRI scans (i.e. after Stage 2–4, Stage 4–3, and the post-learning evaluation period). We scored three points for a trial in which the subject retrieved the food item for the first time in that trial, two points for trials in which they retrieved it at the second attempt, and one point if they needed more than two attempts to retrieve it. The scores were calculated for each food position condition (because the food items were positioned on left and right of the tool shaft in Stage 2–4) in the last five trials in the last three sessions in the steps including the one where the subjects achieved criterion performance. Thirty trials in total (5 (trials) × 2 (both hands) × 3 (sessions)) were analyzed in each evaluation phase. In case of Stage 4–3, 10 trials were calculated for three sessions because there was no specific spatial condition as in the previous stages when the food item was placed to the right or left to the tool. Because a different number of steps were involved in Stage 2–4 (8 preceding steps) and the Stage 4–3 (15 preceding steps), the ratio of these numbers (1.875) was used to multiply the performance scores at the end of the learning and the post-learning period. Thus, the maximum possible score, in which the subject retrieved the food item for the first time in all trials, was 56.25 for each evaluation phase (3 (points) × 10 (trials) × 1.875 (ratio)).

To evaluate the degree of effort in a given trial, we calculated the distance the marmosets moved the rake by video tracking the tool head and measuring the total distance moved from picking up the rake until food retrieval was completed. Because the subjects were free to move the rake until they successfully retrieved the food item, the distance moved showed considerable trial-by-trial variation.

The mean velocity of the rake head was also calculated by dividing the distance moved by the elapsed time. Data beyond two standard deviations for a given learning stage were excluded from further analysis.

### Procedure for Acquiring Structural MRI

Before the MRI scanning, marmosets were deeply anesthetized with an intramuscularly administered mixture of 0.25 ml ketamine (50 mg/kg, Ketalar; Daiichi Sankyo) and 0.04 ml xylazine (5 mg/kg, Selactar; Bayer). Once anesthetized, marmosets were administrated a mixture of oxygen and isoflurane (Abbott Laboratories) with a concentration of 2.5% into a tracheal intubation tube at a constant rate by using an artificial respirator (SN-480-7; Shinano). They were then transferred to a plastic head holder (Qualita) and secured with ear bars in the external auditory meatus. MRI scans were performed using a 7-T Biospec 70/16 MRI scanner (Bruker biospin) equipped with actively shielded gradients that had a maximum strength of 700 mT/m. The inner diameter of the integrated transmitting and receiving coil was 62.0 mm. Each scan took approximately 1 h 40 min, during which the marmoset’s physiological condition was continuously monitored by electrocardiogram, transcutaneous pulse oximetry estimates O_2_ saturation, and skin and rectal temperature.

High-resolution T1-weighted images were acquired with an optimized magnetization-prepared rapidly acquired gradient-echo (MP-RAGE) sequence (time to repetition, 13 ms; time to echo, 3.8 ms; time to inversion, 1500 ms; time to delay, 3700 ms; matrix size, 256 × 256 × 128; field of view, 51.2 × 51.2 × 25.6 mm; number of segments, 4; number of averages, 2) and had an isotropic voxel size of (0.2 mm)^3^ in about 1 h 40 min^28^. The T1-weighted images were used for the VBM analysis.

### VBM and Statistical Analysis

Seven scanning sessions were carried out in each marmoset. As shown in Fig. 1b, two scans were performed in the pre-learning evaluation period, one at the mid-point of the learning protocol, one after marmosets had mastered the task, and three in the post-learning evaluation period (SI 1 for the reasons of timing and number of scans). The timing of the scans during the learning period was dependent on the actual performance of the individual subjects: the scans were conducted when the subjects had completed specific stages of the task (for example, Stages 2 and 4). Preprocessing for VBM were carried out in SPM8 (Wellcome Trust Centre for Neuroimaging, UCL Institute of Neurology, London) running under Matlab (MathWorks). VBM is optimized when images are segmented into gray matter, white matter, and cerebrospinal fluid. To average data across different brains, spatial normalization of the brains to a common template is required^29^. The modulated normalization, multiplied by the Jacobian determinants derived from spatial normalization, was applied to all gray and white matter images to preserve tissue volume. Smoothing was applied using an isotropic Gaussian kernel of 1.0 mm. This kernel size was justified by the small voxel size and relatively small size of the brain^30^.

The design matrix for the group analysis modeled 28 conditions (7 scans × 4 marmosets)^6^. We performed a t-test for a parametric effect of performance score and the rake distance moved using a contrast (mixture) of session-specific parameter estimates. The t-test identified whether the linear contrast of coefficients (describing the relation between tissue volume and performance score or rake distance moved) was significantly different from zero. The performance score and the distance moved were those achieved during the session immediately before each MRI session. The scores of the first two sessions were set to zero because the marmosets could not perform the task at this points. The score was mean-centered within each marmoset to preclude testing for between-subject differences in tissue volume that were not related to score.

The regions of significant tissue volume change were correlated with performance score and rake distance moved, identified in group and individual analysis across marmosets. These regions were first identified by using a statistical threshold of p < 0.05 after correction for multiple comparisons across the whole brain at the voxel level. The threshold was then lowered to p < 0.001 uncorrected across the group to provide a fuller picture of the results. Coordinates and Z scores of cluster peaks and number of contiguous voxels at p < 0.05 (corrected) were reported across all four marmosets. In addition to Z scores of group analysis, we obtained t-values of each subject using the same coordinates and converted to Z scores using the spm_t2z algorithm in SPM. To illustrate the course of the structural changes in the regions identified as highly significant in the above analysis, we extracted the tissue volume from spherical ROI (1.6 mm diameter) with its center at peak voxel in each of the identified regions. These data were also used to calculate the rate of tissue volume change per week in the different stages of the task.

## Additional Information

**How to cite this article**: Yamazaki, Y. *et al.* Neural changes in the primate brain correlated with the evolution of complex motor skills. *Sci. Rep.* **6**, 31084; doi: 10.1038/srep31084 (2016).

## Supplementary Material

Supplementary Information

## Figures and Tables

**Table 1:** The regions of significant gray matter change in V2, V3, SC, and Acb regions correlated with performance score and rake distance moved, identified in group and individual analysis across marmosets.

	Regions						Individual Z scores			
							Coordinates			Group		Subjects			
		x	y	z	Cluster size	Z score						36	54	66	70
Performance	V3 right	9.8	−5.0	9.0	140	5.10	3.56*	N.S.	N.S.	3.45*
	V3 left	−9.4	−7.6	8.6	1289	5.79	N.S.	4.10*	5.23	3.30*
	V2 left	−8.4	−8.4	7.0	1289	6.21	3.32*	3.39 *	5.92	3.73*
	Acb right	0.5	11.5	9.0	132	5.05	3.30*	3.45*	N.S.	3.98*
	Acb left	−1.9	12.1	9.3	30	4.87	N.S.	5.06	N.S.	4.05*
	SC right	0.3	−0.7	10.9	1487	5.68	3.25*	4.64*	4.45*	4.57*
	SC left	−0.5	−0.5	10.9	1487	5.64	N.S.	N.S.	4.29*	4.51*
Distance Moved	Acb right	0.7	9.0	7.2	5287	6.24	N.S.	6.12	N.S.	5.01
	Acb left	−1.3	10.6	9.3	5287	5.83	3.56*	4.83	N.S.	5.04
	SC right	0.1	−0.9	10.9	1460	5.63	3.37*	4.69*	5.03	3.87*
	SC left	−0.5	−2.1	9.5	1460	5.02	3.46*	4.00*	4.01*	3.66*

Regions were first identified by using a statistical threshold of p < 0.05 after correction for multiple comparisons across the whole brain at the voxel level. The threshold were then lowered to p < 0.001 uncorrected (indicated by*) across the group. Coordinates and number of contiguous voxels (cluster size) at p < 0.05 (corrected) are reported across all four marmosets. N.S. indicates not significant even when the statistical threshold was lowered to p < 0.001 uncorrected.
