# Supplementary material for: Neural changes in the primate brain correlated with the evolution of complex motor skills
Source: Sci Rep. 2016 Aug 8;6:31084. doi: 10.1038/srep31084 (PMC4976342; doi:10.1038/srep31084)
Supplement: Supplementary Information [file srep31084-s1.pdf]

Supplementary Materials for

Neural changes in the primate brain correlated with the evolution of complex motor skills

Authors: Y. Yamazaki, K. Hikishima, M. Saiki, M. Inada, E. Sasaki, R. Lemon, C. J. Price, H. Okano*, and A. Iriki*

* Correspondence to: Atsuhi Iriki (iriki@brain.riken.jp); Hideyuki Okano (hidokano@a2.keio.jp)

This PDF file includes:

Supplementary Information. SI1 to SI3

Figs. S1 to S3

Table S1

Supplementary Information

SI 1 Number of scans per individual subject

We scanned the brains of individual subjects seven times in total: once well before the learning period, once just before learning, once during learning, once just after learning, and three times well after learning was complete. It would have been desirable to scan at more time points during the protocol, as in the previous study using Japanese macaques [1]. Because the facilities for training and scanning were at different locations, it was not possible to transfer them by car for many times to avoid possible health problems caused by the long travel, long anaesthetized sessions, and novel environments, especially after learning was completed.

SI 2 Behavioral characteristics of marmsoets in learning to use a tool

Behavioral data such as performance score, velocity of the rake head and the tracking distance of the rake head were all stable after the last step of Stage 4-3 until the post-learning MRI scans conducted 5 or 6 month later (**Fig. 1b-1d**). Thus, the tool use technology was fully maintained by the marmosets over a long period.

The marmosets engaged in daily sessions each of which lasted for 20 minutes. Because we never imposed any food and water control, or any physical restraint such as a primate chair, progress in acquiring use of the tool was completely dependent on the subjects' mood or motivation to participate in the session. As a result, the number of trials conducted per session varied considerably. They usually worked for about 30 to 80 trials per session, but in some cases, they refused to work at all. Such self-paced participation is one of the critical differences in the experimental study of the two species. Acb is known to be related to the reward system, especially when the reward

is dependent on the degree of effort paid: the more effort was needed, the more the Acb was activated [2]. Additionally, the dorsal and ventral striatum seems to be functionally divided from each other in terms of the sources of motivation in human subjects: while activation was stronger in ventral striatum including Acb, ventral caudate nucleus, and ventral globus pallidus when human subjects executed the task skillfully than less skillfully, dorsal striatum such as dorsal caudate was more activated when the reward option was monetary than non-monetary [3]. These results obtained in humans could be relevant to those found in the common marmoset, considering the positive correlation between increase of the gray matter of bilateral Acb and the rake distance moved, as shown in **Table 1**. Thus, the reason why non-restrained animals were eager to participate in learning tool use could have been related to a sort of intrinsic motivation to join the rewarded and social situation, which would be connected to their original pro-social dispositions, observed frequently in captive and wild situations [4], even when the interaction is cross-species [5].

SI 3 Posture and Hand use in the Tool-Use Task

Because the marmosets were not restrained in a primate chair, they were free to take any postures inside the test chamber to retrieve the food item. Mostly, they were in a quadrupedal posture to maximize their reach towards the food items. During the entire period of the study (which consisted of four main stages with 15 minor steps in total), all marmosets adopted a quadrupedal posture.

As was found in the previous study [6], except for the last step of Stage 4 (Stage 4, step 3, “Stage 4-3” thereafter) in which the rake head and the food item were placed symmetrically in a straight line, the marmosets used either of the hands depending on

the relationship between the food item and the tool. When the food item was located in area to the right of the tool shaft (viewed from the marmoset's perspective), they used left hand. When the food item was located to the left of the tool shaft, they used the right hand. In this final stage, marmosets preferred adduction to abduction of the arm when they made an arc to retrieve food items. In the last step, however, there was no such left-right bias in the relationship between the food item and the tool shaft, each marmoset exhibited an individual hand preference during the trials. The number trials in which the marmoset first used its left hand to touch the rake in the criterion trials (i.e. consecutive three successful retrieval of the food item) in Stage 4-3 by 36, 54, and 70 was three, and that of 66 was two. These tendencies of hand preference remained similar in the post-learning evaluation sessions (Post) conducted several months after the end of the learning of Stage 4-3 (**Table S1**).

References

1. Quallo, M. M. et al. Gray and white matter changes associated with tool-use learning in macaque monkeys. *Proc. Natl. Acad. Sci. U. S. A.* **106**, 18379-18384 (2009).

2. Salamone, J. D. & Correa, M. The mysterious motivational functions of mesolimbic dopamine. *Neuron* 76, 470-485 (2012).

3. Lutz, K., Pedroni, A., Nadig, K., Luechinger, R. & Jäncke, L. The rewarding value of good motor performance in the context of monetary incentives. *Neuropsychologia* **50**, 1739-1747 (2012).

4. Burkart, J. M., Fehr, E., Efferson, C. & van Schaik, C. P. Other-regarding preferences in a non-human primate: Common marmosets provision food altruistically. *Proceedings of the National Academy of Sciences* **104**, 19762-19766 (2007).

5. Izar, P. et al. Cross-genus adoption of a marmoset (*Callithrix jacchus*) by wild capuchin monkeys (*Cebus libidinosus*): case report. *Am. J. Primatol.* **68**, 692-700 (2006).

6. Yamazaki, Y. et al. Tool-use learning by common marmosets (*Callithrix jacchus*). *Exp. Brain Res.* **213**, 63-71 (2011).

7. Paxinos, G., Watson, C., Petrides, M., Rosa, M. G. P. & Tokuno, H. in *The marmoset brain in stereotaxic coordinates* (Elsevier Inc., 2012).

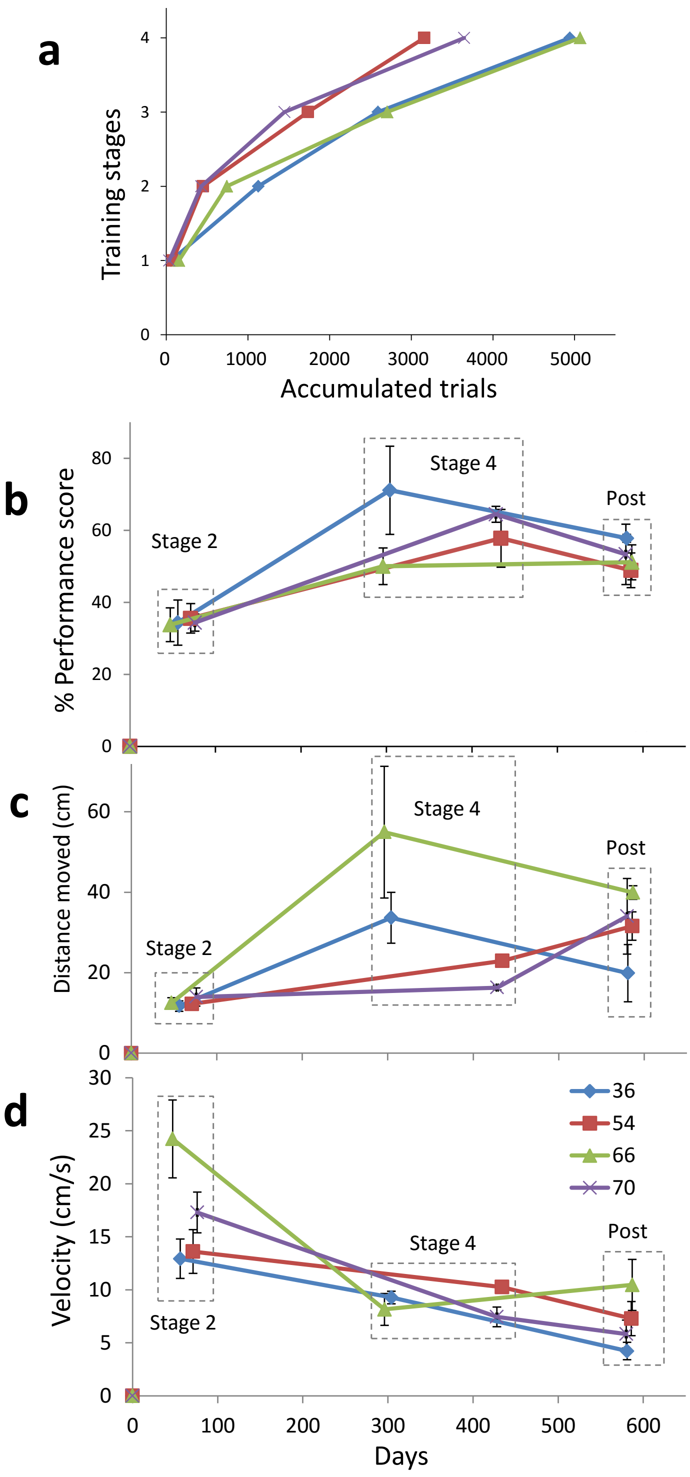

Fig. S1 Behavioral indices of the individual subjects during the tool use learning: (a) the accumulated number of trials to achieve the criterion in each stage in each subject, (b) % performance score, (c) distance moved by the rake head (cm), and (d) velocity of the rake head (cm/s). Error bars show SE.

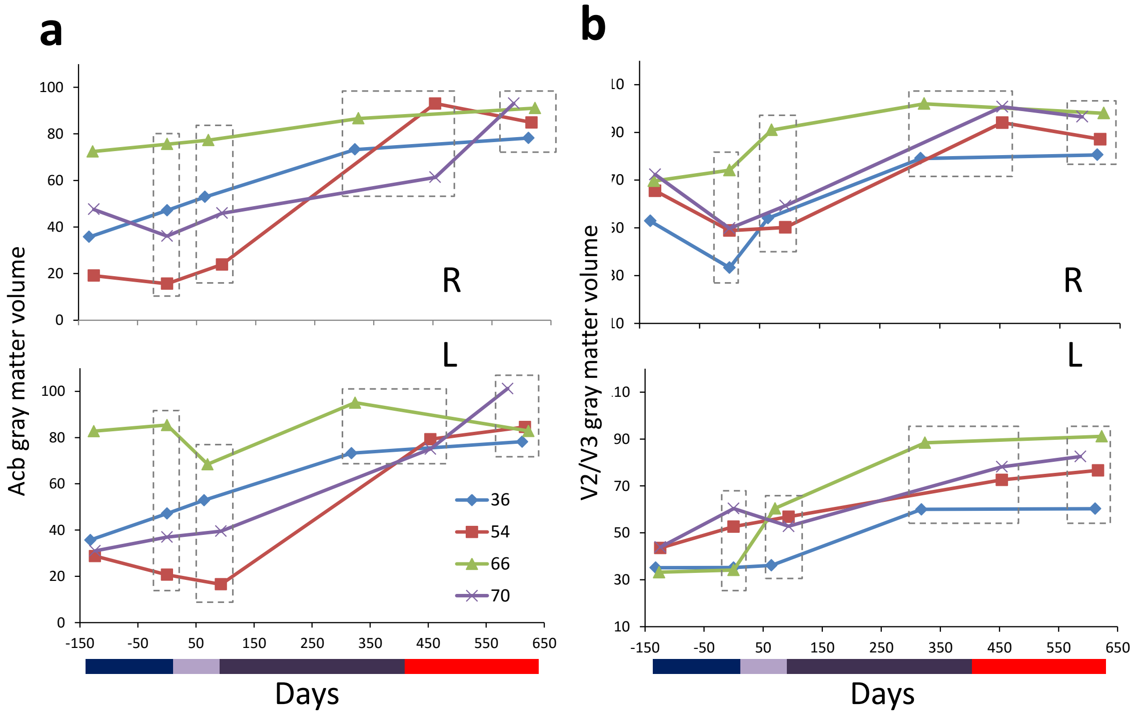

Fig. S2 Time course of individual gray matter volume changes in the right (upper panels, "R") and the left (lower panels, "L") hemispheres in peak voxels in Acb (a) and V2/V3 (b). It is averaged in four marmosets as a function of the experimental phases in individual subjects, together with the averaged data, with SE. A color bar on the bottom of the figures corresponds to the experimental phases of pre-learning evaluation (dark blue), Stage 1 to 2 (light purple), Stage 3 to 4 (dark purple), and post-learning evaluation (red).

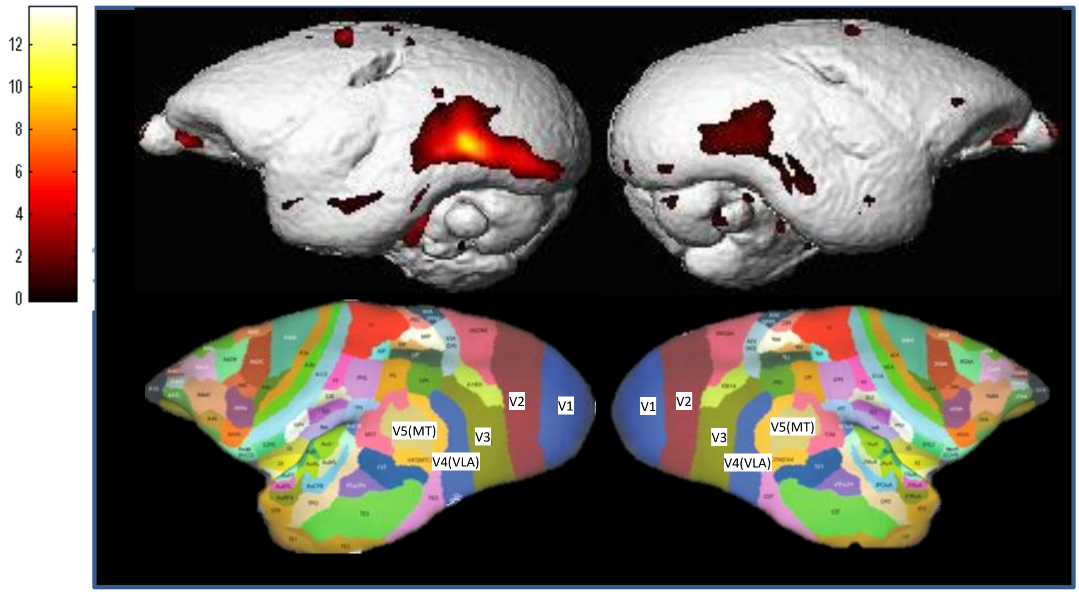

Fig. S3 Surface cortical map of subject 54 with comparison of the area definition by the atlas (reproduced from [7] with permission). Volume increase on the posterior part of the brain shows lateral extrastriate cortex, extended from V2, V3, and MT.

Table S1 The number of trials in which the marmoset used either left or right hand to first touch the rake during trials in Stage 4-3 and in the post learning period in individual subjects. After an interval of more than five months the hand use was stabilized especially in marmosets 36 and 70.

	Stage 4-3		Follow UP	
Subjects	Left	Right	Left	Right
36	3	0	3	0
54	3	0	2	1
66	2	1	1	2
70	3	0	3	0
